# Supplementary material for: Plasma Membrane H+-ATPase SmPHA4 Negatively Regulates the Biosynthesis of Tanshinones in Salvia miltiorrhiza
Source: Int J Mol Sci. 2021 Mar 25;22(7):3353. doi: 10.3390/ijms22073353 (PMC8037235; doi:10.3390/ijms22073353)
Supplement: Supplementary file 1 [file ijms-22-03353-s001.pdf]

Table S1 Primers used in this test for real-time quantitative PCR

Primer name	For	Rev
SmPHA1	GATTGTCGGTTCGTGGCCTT	GCCTGGTCGTCCAAACACAT
SmPHA2	TCGAGGCTGGAGAAGCAAGA	TCCGGTGCACCTTTGCTAAC
SmPHA3	ACCAGGACGCCATAGATGCT	CACAGCATGAACCCTTCGCT
SmPHA4	CCCTGCTGATGCTCGTCTTC	CCATACCAACGGCGATGGAG
SmPHA5	CACGAAACGGATGACAGCCA	CCCGAGCTGCGAGAAGAATC
SmPHA6	AAGAAGCCGCCATCCTTGTG	GGCAATGACGACAGCCTCAA
SmPHA7	GGAAGTTCCAGAGGGACGGA	CACACCGTCACCAGTCATCC
SmPHA8	TACGTGCCACTCGACCTCAT	TGCAATCTCAGCGCGTTTCT
SmPHA9	TGCAAATGGAGGAGGCAAGC	CAGTGGATCGCCATCAAGCA
SmActin	GGTGCCCTGAGGTCCTGTT	TGGACCCTCCAATCCAGACA
SmDXS2	CTCACGGTCGCATTGCATCAT	CGCTTTCGTCTCGTTTAGGGA
SmAACT1	TGAAGGACGGACTCTGGGATGT	CCTTGTCAACAATGGTGGATGG
SmKSL1	TGGAAACAGTGTGACCCTTCTGCT	GCTTGCATACAAATAACACCCAATCCT
SmCYP76AH1	ACGCATCACTTCACCCATCTCAA	TTGCCGACTCATCCACGAT

Table S2 Primers used in this test for plasmids construction or identification

Primer name	Sequence
SmPHA4-attB-for	GGGGACAAGTTTGTACAAAAAAGCAGGCTATGTGGAATCCACTCTCTTGG
SmPHA4-attB-Rev	GGGGACCACTTTGTACAAGAAAGCTGGGTTTAGACGGTGTAATGTTGCTG
RNAiSmPHA4-attB-For	GGGGACAAGTTTGTACAAAAAAGCAGGCTGGTTTTGCTGGGGTGTTTC
RNAiSmPHA4-attB-Rev	GGGGACCACTTTGTACAAGAAAGCTGGGTAGGCAATGGAGATGGCTTC
RNAi-dsRed-for	GGGGACAAGTTTGTACAAAAAAGCAGGCTCATCCCCGACTACAAGAAG
RNAi-dsRed-rev	GGGGACCACTTTGTACAAGAAAGCTGGGTTGGTGTAGTCCTCGTTGTG
rolB-For	GCTCTTGCAGTGCTAGATTT
rolB-Rev	GAAGGTGCAAGCTACCTCTC
rolC-For	CTCCTGACATCAAACTCGTC

rolC-Rev	TGCTTCGAGTTATGGGTACA
pK7-NPTIIF	ACGTTGTCACTGAAGCGGGAAGG
pK7-NPTIIR	GGCGATACCGTAAAGCACGAGGAA
p35S-for	GACGCACAATCCCACTATCC
